# A glycolysis-based three-gene signature predicts survival in patients with lung squamous cell carcinoma

**DOI:** 10.1186/s12885-021-08360-z

**Published:** 2021-05-27

**Authors:** Guichuan Huang, Jing Zhang, Ling Gong, Yi Huang, Daishun Liu

**Affiliations:** 1 Department of Pulmonary and Critical Care Medicine, The First People’s Hospital of Zunyi (The Third Affiliated Hospital of Zunyi Medical University), No 98 Fenghuang Road, Huichuan District, Zunyi, 563000 China; 2 Department of Pulmonary and Critical Care Medicine, Affiliated Hospital of Zunyi Medical University, Zunyi, Guizhou China

**Keywords:** Lung cancer, Glycolysis, Prognosis, Gene signature

## Abstract

**Background:**

Lung cancer is one of the most lethal and most prevalent malignant tumors worldwide, and lung squamous cell carcinoma (LUSC) is one of the major histological subtypes. Although numerous biomarkers have been found to be associated with prognosis in LUSC, the prediction effect of a single gene biomarker is insufficient, especially for glycolysis-related genes. Therefore, we aimed to develop a novel glycolysis-related gene signature to predict survival in patients with LUSC.

**Methods:**

The mRNA expression files and LUSC clinical information were obtained from The Cancer Genome Atlas (TCGA) dataset.

**Results:**

Based on Gene Set Enrichment Analysis (GSEA), we found 5 glycolysis-related gene sets that were significantly enriched in LUSC tissues. Univariate and multivariate Cox proportional regression models were performed to choose prognostic-related gene signatures. Based on a Cox proportional regression model, a risk score for a three-gene signature (HKDC1, ALDH7A1, and MDH1) was established to divide patients into high-risk and low-risk subgroups. Multivariate Cox regression analysis indicated that the risk score for this three-gene signature can be used as an independent prognostic indicator in LUSC. Additionally, based on the cBioPortal database, the rate of genomic alterations in the HKDC1, ALDH7A1, and MDH1 genes were 1.9, 1.1, and 5% in LUSC patients, respectively.

**Conclusion:**

A glycolysis-based three-gene signature could serve as a novel biomarker in predicting the prognosis of patients with LUSC and it also provides additional gene targets that can be used to cure LUSC patients.

**Supplementary Information:**

The online version contains supplementary material available at 10.1186/s12885-021-08360-z.

## Background

Lung cancer is the leading cause of cancer-related mortality worldwide. There are two clinical subtypes for lung cancer: non-small cell lung cancer (NSCLC) (approximately 85% occurrence), and small cell lung cancer (SCLC) (approximately 15% occurrence) [1]. Based on pathological and molecular features, NSCLC is divided into the following major subtypes: lung squamous cell carcinoma (LUSC), lung adenocarcinoma (LUAD), and large cell lung cancer [2]. Recent advances in targeted treatments, such as epidermal growth factor receptor (EGFR) kinase inhibitors, have increased the overall survival (OS) of patients with LUAD [3]. However, no specific biomarkers or relatively optimal targeted therapies have been identified for LUSC patients, and the 5-year survival rate of LUSC is less than 20% [4]. Therefore, it is necessary to explore specific diagnostic and prognostic biomarkers for LUSC.

Energy metabolism reprogramming is a process that promotes cancer cell growth and proliferation via adjustment of energy metabolism, and it has been regarded as an emerging hallmark of cancer [5]. Under aerobic conditions, normal cells obtain energy through mitochondrial oxidative phosphorylation. Under anaerobic conditions, the cells obtain energy via glycolysis instead of oxygen-consuming mitochondrial metabolism [6]. Glycolysis, also known as the Warburg effect, is often observed in human cancer cells, in which the cancer cells favor glucose metabolism via glycolysis even in the presence of oxygen [7]. This phenomenon is a unique energy metabolism that exists in cancer cells. In recent years, many biomarkers for LUSC have been discovered, including glycolysis-associated genes such as kininogen 1 (KNG1) [8] and tripartite motif-containing protein 59 (TRIM59) [9].

With the development of high-throughput sequencing, various patient genome databases have been constructed, which enables us to acquire a deep understanding of genomic changes. Based on database mining, an increasing number of biomarkers have been identified that are related to the survival of patients with cancer [10, 11]. However, a single gene cannot be used to obtain satisfactory predictive effects. A multigene prognostic model from an original tumor biopsy can guide clinicians to choose more effective treatment strategies. Thus, a signature based on multigene expression associated with glycolysis should be established to predict the prognosis of LUSC patients.

In the present study, we used a genome-wide analysis of LUSC patient mRNA expression profiles from The Cancer Genome Atlas (TCGA) to construct a glycolysis-related gene signature that could effectively predict the prognosis in LUSC patients.

## Methods

### Patient dataset

The mRNA expression profiles of LUSC patients and their corresponding clinical information were obtained from the TCGA database (https://portal.gdc.cancer.gov/). A total of 502 LUSC samples and 49 tumor-adjacent normal samples were downloaded from TCGA. Each tumor specimen was approximately 1 cm^3^ in size and weighed between 100 mg and 200 mg, in general [12]. Then, we downloaded the clinical data for LUSC (including 504 patients) from TCGA. There were 501 matched LUSC patients between the mRNA expression files and the clinical information. Therefore, a total of 501 tumor samples and 49 tumor-adjacent normal samples were included in our study. Clinical information, including age, sex, American Joint Committee on Cancer (AJCC) stage, T, M, N, survival time, and survival status were included in the present study (Table 1). Additional information regarding surgically extracted LUSC can be seen in the TCGA collection protocols [12, 13].

**Table 1:** Clinical characteristic of LUSC (*n* = 501) from TCGA database

Clinical characteristic	N	%
**Age**		
≤ 65	190	37.9
> 65	302	60.3
NA	9	1.8
**Sex**		
Female	130	25.9
Male	371	74.1
**AJCC stage**		
I	3	0.6
IA	90	18.0
IB	151	30.1
II	3	0.6
IIA	65	13.0
IIB	94	18.8
III	3	0.6
IIIA	63	12.6
IIIB	18	3.6
IV	7	1.4
NA	4	0.8
**T**		
T1	114	22.7
T2	293	58.5
T3	71	14.2
T4	23	4.6
**N**		
N0	319	63.7
N1	131	26.1
N2	40	8.0
N3	5	1.0
NA	6	1.2
**M**		
M0	411	82.0
M1	7	1.4
NA	83	16.6
**Survival time**		
Reported	495	98.8
NA	6	1.2
**Survival status**		
Alive	286	57.1
Dead	215	42.9

*Abbreviations*: *LUSC* Lung squamous cell carcinoma, *TCGA* The cancer genome atlas, *NA* Not available, *AJCC* American joint committee on cancer

### Gene set enrichment analysis (GSEA)

GSEA was performed to determine whether there were significant differences in the identified gene sets between the LUSC and normal groups. The expression levels of 443 glycolysis-related genes were analyzed in LUSC samples and in adjacent non-cancerous tissues. A normalized *p* value less than 0.05 was considered statistically significant.

### Prognostic analysis

We conducted univariate Cox proportional hazard regression analysis to determine the relationship between glycolysis-related genes and OS in LUSC patients. If *p* < 0.01, the corresponding glycolysis-related genes were retained and regarded as candidate prognostic genes for LUSC. Then, a multivariate Cox proportional hazards regression analysis was performed among the pooled candidate prognostic glycolysis-related genes to establish the prognostic model. These analyses were performed with the use of the R package for survival.

### Statistical analysis

The selected mRNAs were divided into the risky [hazard ratio (HR) > 1] and protective (0 < HR < 1) types. Based on a linear combination of the expression level of filtered mRNAs weighted by the regression coefficient (β), the formula for the risk score is illustrated as follows: Risk score = expression of gene 1 × β1+ expression gene 2 × β2 + … + expression of gene n × βn. β represents the regression coefficient of the corresponding gene obtained from the multivariate Cox regression model. According to the median value of the risk score, patients were divided into high-risk or low-risk groups. Kaplan-Meier curves and log-rank tests were utilized to validate the prognostic significance of the risk score.

Student’s *t*-test or the Mann-Whitney *U*-test was conducted to explore the differential expression of selected genes in LUSC tissues and adjacent normal tissue. If the expression data of selected genes followed a normal distribution, Student’s *t*-test was used to analyze differences between LUSC tissues and adjacent normal tissue; otherwise, the Mann-Whitney *U*-test was utilized. Filtered gene alterations in LUSC were explored using the cBioPortal database (http://www.cbioportal.org/). All statistical analyses were performed using the R language and environment for statistical computing (R version 3.6.3). Visualization of results was performed using R software.

## Results

### Initial screening of genes by GSEA

**Fig. 1:**
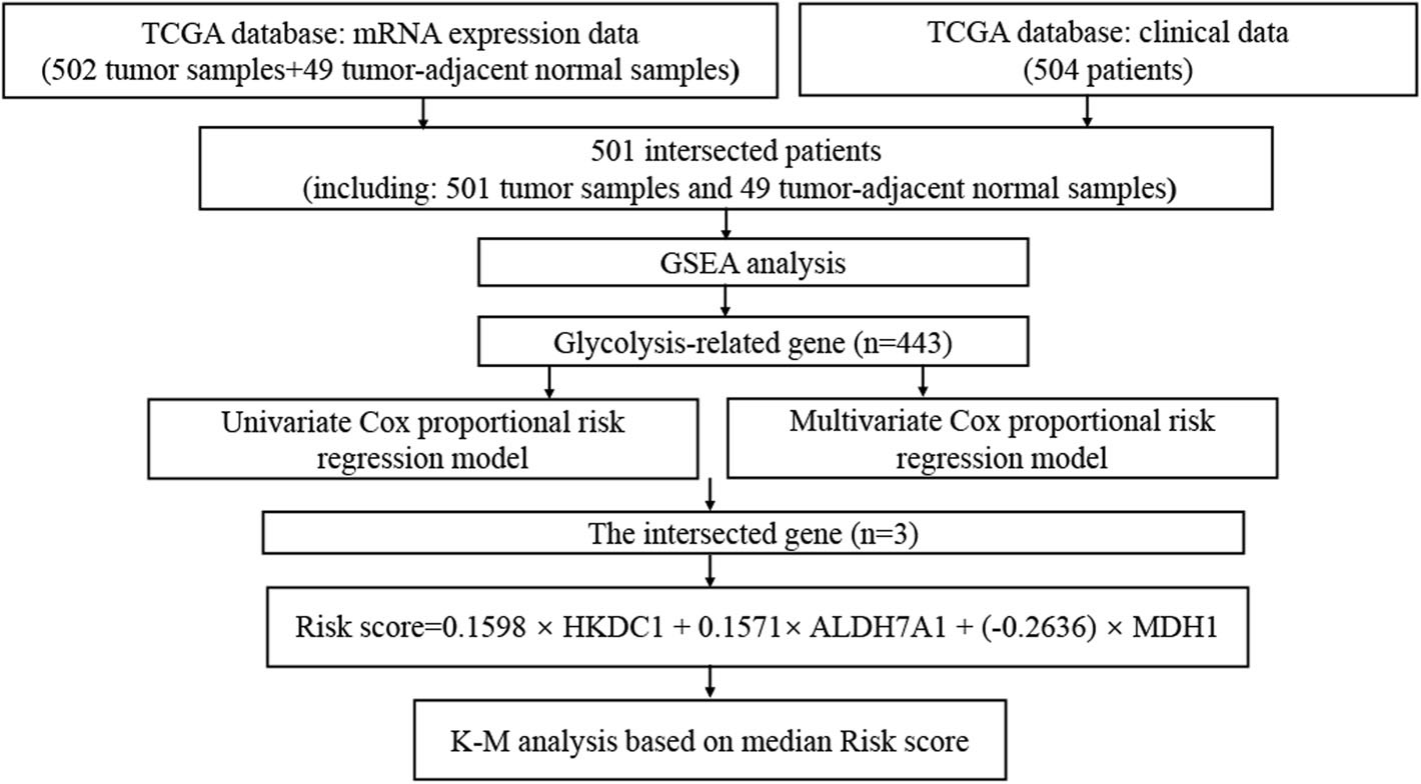
The flow chart of study

**Fig. 2:**
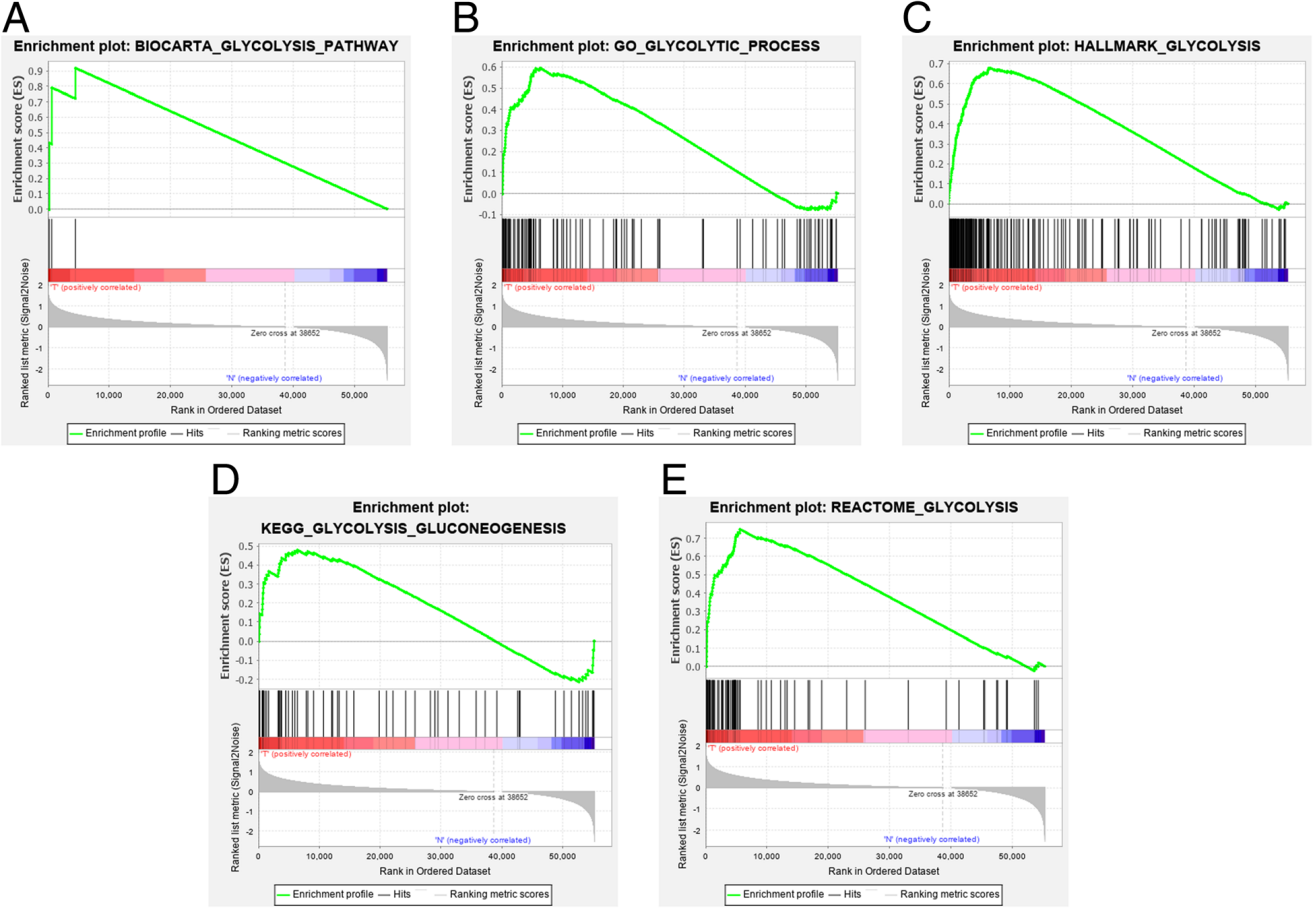
GSEA results for enrichment plots of five gene sets which were significantly differentiated between in LUSC and normal tissues. **A**, BIOCARTA_GLYCOLYSIS_ PATHWAY; **B**, GO_GLYCOLYTIC_PROCESS; **C**, HALLMARK_GLYCOLYSIS; **D**, KEGG_GLYCOLYSIS_GLUCONEOGENESIS; **E**, REACTOME_ GLYCOLYSIS. Abbreviations: GSEA, gene set enrichment analysis; LUSC, lung squamous cell carcinoma

The mRNA expression data set and clinical information for 501 patients with LUSC were obtained from the TCGA database (Fig. 1). We found five glycolysis-related gene sets in the Molecular Signatures Database v7.0, including the (1) BIOCARTA_GLYCOLYSIS_ PATHWAY, (2) GO_GLYCOLYTIC_PROCESS, (3) HALLMARK_GLYCOLYSIS, (4) KEGG_GLYCOLYSIS_GLUCONEOGENESIS, (5) REACTOME_ GLYCOLYSIS. We performed GSEA to explore whether there were significant differences between LUSC and normal tissues in the identified gene sets. We found that these 5 gene sets were significantly enriched (Fig. 2 and Table 2). Then, we collected 443 genes from 5 gene sets for further analysis.

**Table 2:** Gene set enriched in LUSC

Gene sets follow link to MSigDB	Size	NES	NOM ***p***-val	FDR q-val
BIOCARTA_GLYCOLYSIS_ PATHWAY	3	1.43	0.029	0.029
GO_GLYCOLYTIC_PROCESS	106	2.01	< 0.0001	< 0.0001
HALLMARK_GLYCOLYSIS	200	2.29	< 0.0001	< 0.0001
KEGG_GLYCOLYSIS_GLUCONEOGENESIS	62	1.56	0.028	0.028
REACTOME_ GLYCOLYSIS	72	2.28	< 0.0001	< 0.0001

*Abbreviations*: *LUSC* Lung squamous cell carcinoma, *MSigDB* Molecular signatures database, *NES* Normalized enrichment score, *NOM p-val* Nominal *p*-value, *FDR q-val* False discovery rate q-value

### Identification of glycolysis-related genes associated with patient survival

First, univariate Cox proportional hazard regression analysis was conducted for 443 genes that were significantly enriched in LUSC samples from the GSEA. A total of 4 genes were obtained that were significantly correlated with patient survival (*p* < 0.01). Next, we performed multivariate Cox regression analysis to further explore the association between the 4 mRNA expression profiles and the OS of patients.

Finally, 3 genes, hexokinase domain-containing protein 1 (HKDC1), aldehyde dehydrogenase 7A1 (ALDH7A1), and malate dehydrogenase 1 (MDH1), were included to construct a prognostic model. As shown in Table 3, two of the three genes were verified as independent prognostic markers in LUSC. Among the three genes, one gene (MDH1) was considered as a protective factor according to 0 < HR < 1, whereas the remaining two genes (HKDC1 and ALDH7A1) might be prognostic risk factors with their HR > 1.

**Table 3:** Details of three genes for constructing the prognostic model

Gene	Ensemble ID	Location	HR (95%CI)	Coefficient	***p*** value
HKDC1	ENSG00000156510	chr10: 69,220,303-69,267,559	1.1733	0.1598	0.0446
ALDH7A1	ENSG00000164904	chr5: 126,531,200-126,595,390	1.1701	0.1571	0.0097
MDH1	ENSG00000014641	chr2: 63,588,609-63,607,197	0.7682	−0.2636	0.0559

*Abbreviation*: *HR* Hazard ratio

Subsequently, we explored the alterations of three selected genes in 501 LUSC samples using the cBioPortal database. The results showed that the rates of genomic alterations in the HKDC1, ALDH7A1, and MDH1 genes were 1.9, 1.1, and 5%, respectively (Supplementary Figure 1).

**Fig. 3:**
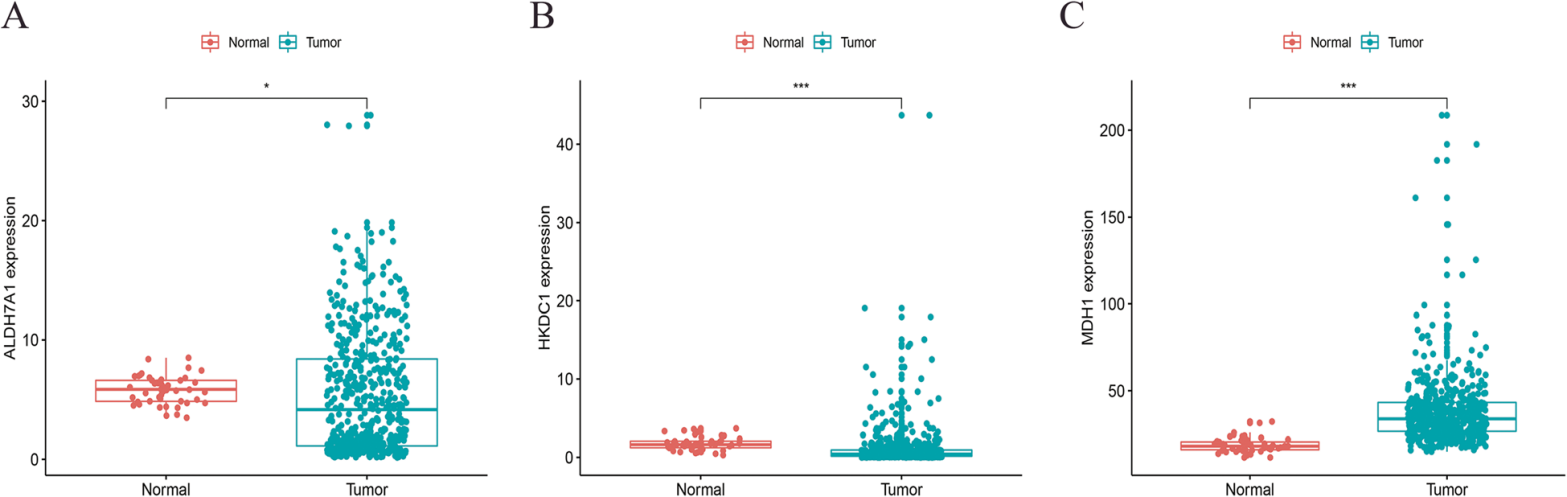
Differential expression of three genes in the normal tissues (*n* = 49) and tumor tissues (*n* = 501). (* *p* < 0.05, **p < 0.01, ***p < 0.001). **A**, ALDH7A1; **B**, HKDC1; **C**, MDH1

The expression level of the three genes was measured between adjacent normal tissues and LUSC tissues. We found that all three genes were upregulated in LUSC tissues compared with normal tissues (Fig. 3).

### Construction of the three-gene signature to predict patient prognosis

To predict patient prognosis using glycolysis-related gene expression, a prognostic risk model was developed based on the regression coefficients of the multivariate Cox regression model to weight the expression level of each gene in the three-gene signature: risk score = 0.1598 × expression value of HKDC1 + 0.1571 × expression value of ALDH7A1 + (− 0.2636) × expression value of MDH1. Because 6 of 501 patients lacked survival time data, a total of 495 patients were included in the survival analysis. The clinical information for 495 patients is listed in Supplementary Table 1.

**Fig. 4:**
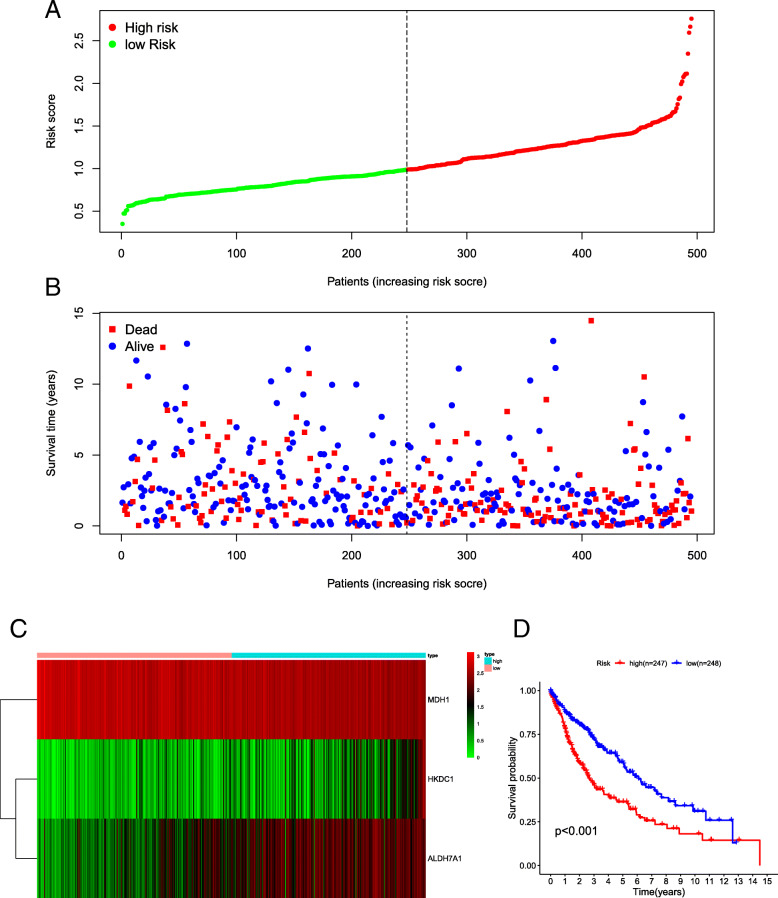
A risk of three-gene signature predicted the overall survival in patients with LUSC. **A**, Distribution of risk score per patient; **B**, Survival status of each patients; **C**, A heatmap of three genes expression profile; **D**, Kaplan-Meier survival curve analysis for LUSC patients divided into the high-risk and low-risk groups. Abbreviation: LUSC, lung squamous cell carcinoma

**Fig. 5:**
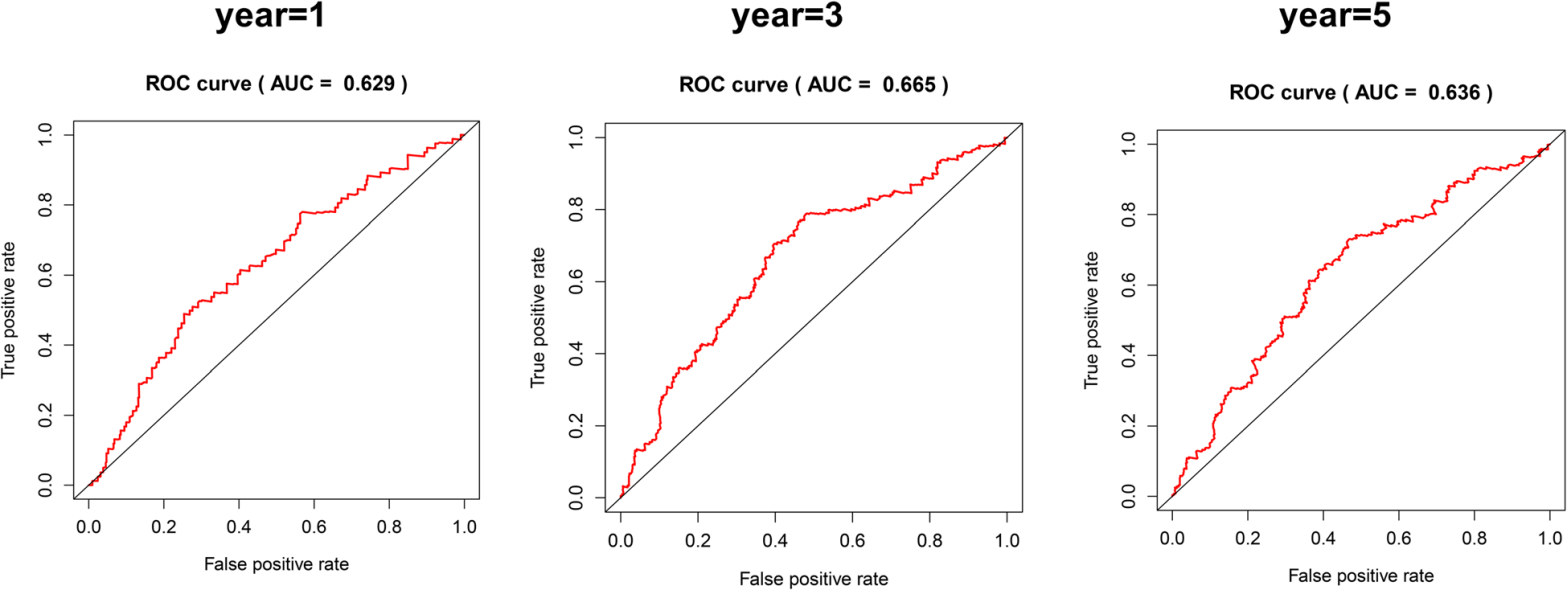
The time-independent ROC curve of the risk score for prediction the 1, 3, 5-year overall survival

According to the risk score formula, patients were classified into the high-risk (*n* = 247) or the low-risk group (*n* = 248) with a median value of risk score as a cut-off (Fig. 4A). The survival time for each patient is shown in Fig. 4B. As shown in Fig. 4D, patients in the high-risk group had shorter survival as compared to the low-risk group (*p* < 0.001). The 3-year and 5-year survival rates of patients in the high-risk group were 45.4 and 35.0%, respectively. However, the 3-year and 5-year survival rates of patients in the low-risk group were 71.9 and 58.1%, respectively. Additionally, a heatmap presents the expression profiles of three mRNAs (Fig. 4C). As the risk score increased in patients with LUSC, the mRNA expression of HKDC1 and ALDH7A1 was obviously upregulated; in contrast, the mRNA expression of MDH1 was downregulated. The area under the receiver operating characteristic (ROC) curve (AUC) for the risk score at 1-, 3-, and 5-year OS was 0.629, 0.665, and 0.636, respectively (Fig. 5).

### Risk score from the three-gene signature is an independent prognostic indicator

Univariate and multivariate Cox regression analysis were performed to evaluate the independent risk factors in patients with LUSC. Several clinicopathological parameters, including age, sex, AJCC stage, T, N, M, as well as risk score were included. The results showed that only the risk score was associated with prognosis in the univariate Cox analysis (HR = 2.553, 95% CI: 1.710–3.811, *p* < 0.0001) (Table 4). In the following multivariate Cox analysis, it was determined that age and risk score were independent prognostic indicators (Table 4). These results indicated that the risk score was reliable in predicting the prognosis of patients with LUSC.

**Table 4:** Univariate and multivariate Cox regression analysis of clinicopathologic factors and glycolysis-related genes signature for OS

Clinical features	Univariate analysis			Multivariate analysis		
	HR	95%CI of HR	***P*** value	HR	95%CI of HR	***P*** value
Age (> 65 vs. <=65)	1.376	1.000–1.892	0.050	1.489	1.078–2.055	0.016
Sex (Male vs. Female)	1.360	0.944–1.958	0.099	1.300	0.901–1.877	0.161
AJCC stage (III-IV vs. I-II)	1.392	0.974–1.989	0.070	1.185	0.716–1.962	0.510
T (T3 + T4 vs. T1 + T2)	1.412	0.973–2.048	0.069	1.335	0.845–2.110	0.216
M (M1 vs. M0)	2.432	0.898–6.591	0.080	2.216	0.768–6.399	0.141
N (N1 + N2 + N3 vs. N0)	1.062	0.780–1.444	0.704	1.087	0.765–1.545	0.642
Risk score (high risk vs. low-risk)	2.553	1.710–3.811	< 0.0001	2.663	1.790–3.962	< 0.0001

*Abbreviations*: *OS* Overall survival, *HR* Hazard ratio

### Validation of the three-gene signature for survival prediction by Kaplan-Meier curve analysis

**Fig. 6:**
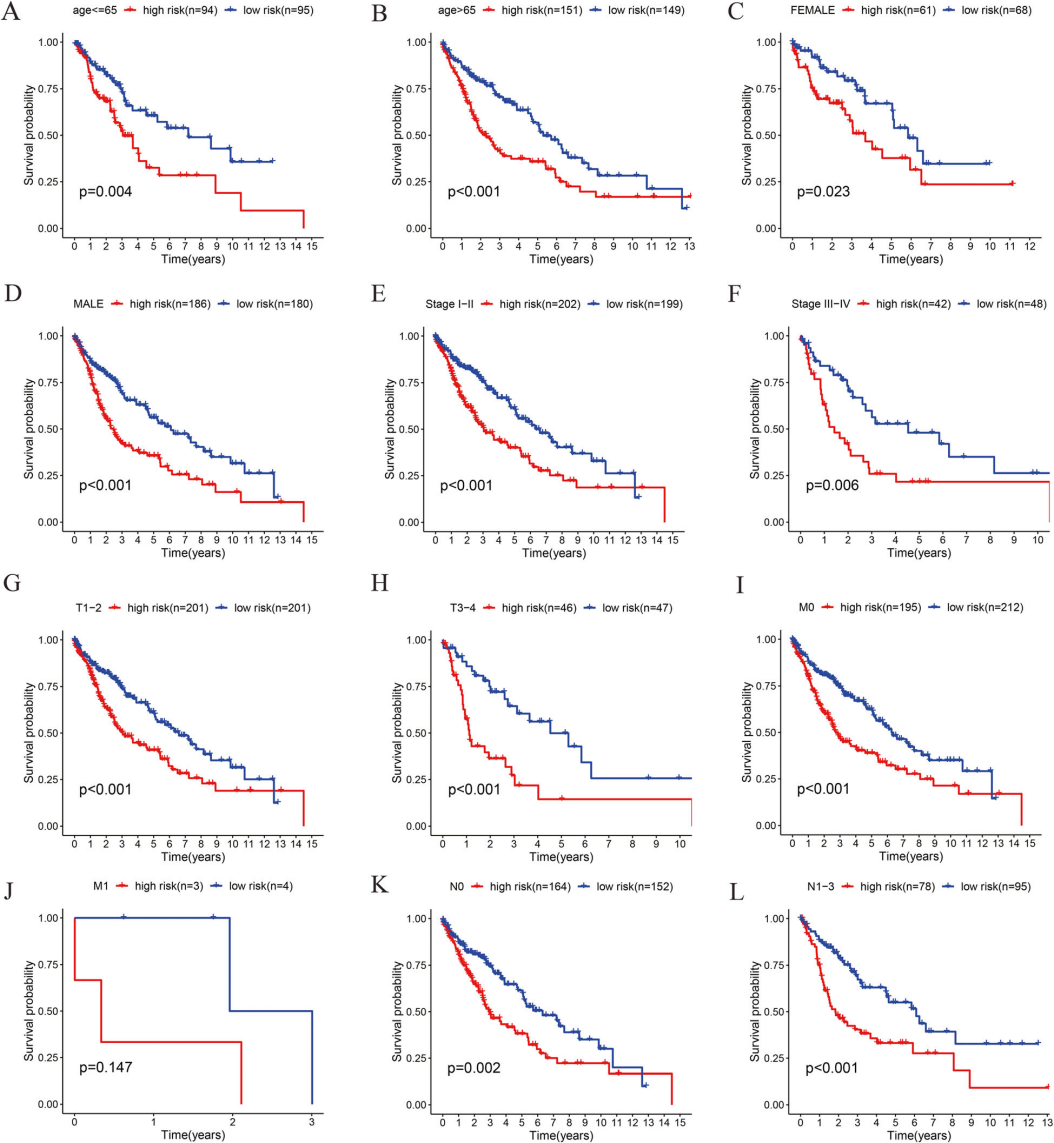
Validation for prognostic value of the risk score in different subgroups. **A**, Subgroup for age ≤ 65; **B** Subgroup for age > 65; **C**, Subgroup for Female; **D** Subgroup for Male; **E**, Subgroup for AJCC stage I-II; **F**, Subgroup for AJCC stage III-IV; **G**, Subgroup for T1 + T2; **H**, Subgroup for T3 + T4; **I**, Subgroup for M0; **J**, Subgroup for M1; **K**, Subgroup for N0; **L**, Subgroup for N1 + N2 + N3. Abbreviation: AJCC stage, American Joint Committee on Cancer stage

To further verify the prognostic value of the risk score of the three-gene signature associated with glycolysis, patients with LUSC were stratified by age (≤65 or > 65), sex (female or male), AJCC stage (I + II or III + IV), T (T1 + T2 or T3 + T4), N (N0 or N1 + N2 + N3), and M (M0 or M1) (Fig. 6). We found no significant difference between high-risk and low-risk in patients with remote tumor metastasis (Fig. 6J). However, in the subgroup of patients without remote tumor metastasis, the risk score for the three-gene signature was still an independent prognostic indicator (Fig. 6I). Additionally, regardless of the age, sex, AJCC stage, T or N, patients in the high-risk group based on the risk score had a poor prognosis when compared to patients in the low-risk group. These findings demonstrated that the three-gene signature effectively predicts the survival of LUSC patients.

## Discussion

Recently, numerous genes have been considered as biomarkers for cancer prognosis, and the clinical significance of the biomarkers has been explored. For example, a study by Tang and his colleagues found that the overexpression of dipeptidyl peptidase 9 (DPP9) was a significant independent factor for poor prognosis in patients with NSCLC [14]. Similarly, Feng et al. [15] reported that high expression of forkhead box Q1 (FoxQ1) was associated with poor prognosis in patients with NSCLC. However, the expression level of single genes can be influenced by multiple factors, and thus, these biomarkers can be unreliable for independent prognosis indications. Therefore, a statistical model based on a combination of multiple genes was used to improve the prediction of prognosis in cancer patients. Studies have shown that a pool of multiple genes was more accurate than a single gene in predicting the prognosis of patients with cancer [16, 17].

In the present study, we obtained mRNA expression profiles for 501 LUSC patients from the TCGA database. We found that 5 glycolysis-related gene sets were significantly enriched in LUSC samples using GSEA. Univariate and multivariate Cox regression analyses were carried out to identify the risk score for the three-gene signature with prognostic value for patients with LUSC. Kaplan-Meier curve analysis indicated that patients with a high risk score had a poor prognosis when compared to patients with a low risk score. Additionally, in the stratified analysis, the risk score for the three-gene signature effectively predicted the prognosis of LUSC patients in all subgroups except for the subgroup of patients with remote tumor metastasis. The reason for this discrepancy might be that the number of patients with remote tumor metastasis was too small (*n* = 7). These results demonstrated that the risk score for the three-gene signature could be used as an independent prognostic indicator for LUSC patients. Moreover, measuring the patient risk score might assist clinicians in choosing optimal therapy methods.

The metabolism of tumor cells is more active than that of normal cells, and therefore, tumor cells require a greater amount of energy to maintain their higher proliferation [18]. Glycolysis and oxidative phosphorylation are the two important metabolic pathways related to energy supply. Glycolysis is a relatively low-energy-providing pathway compared with oxidative phosphorylation. In the 1920s, Warburg found that cancer cells are very active in glycolysis and require a large amount of glucose to obtain ATP for metabolic activities [19]. This aberrant phenomenon of glucose metabolism was called aerobic glycolysis, and is also known as the Warburg effect [19, 20]. There was further research on the main genes and enzymes related to glycolysis to gain understanding of their functions in the metabolism of tumor cells.

In recent years, studies have shown that aerobic glycolysis plays a significant role in tumorigenesis, tumor progression, and metastasis. For example, enolase 1 (ENO1) was proved to promote cell glycolysis, growth, migration, and invasion in NSCLC [21]. Glucose transporter 1 (GLUT1) facilitated increased transport of glucose into cancer cells to maintain an elevated rate of glycolysis under aerobic conditions [22]. A high expression of GLUT1 was significantly associated with a poor prognosis in lung cancer patients [23]. However, no set of glycolysis-related genes for predicting LUSC prognosis has been established.

HKDC1, a recently identified fifth hexokinase, plays an important role in cellular glucose metabolism [24]. Aberrational expression of HKDC1 is associated with various cancers, including colorectal cancer [25], liver cancer [26], and breast cancer [27]. Additionally, Wang and his colleagues reported that HKDC1 was overexpressed in LUAD tissues, and high expression of HKDC1 promoted proliferation, migration, and invasion in LUAD [28]. Thus, HKDC1 can serve as a prognostic biomarker for LUAD patients [28]. The aldehyde dehydrogenase (ALDH) superfamily comprises 19 enzymes that play a vital role in maintaining epithelial homeostasis. ALDH activity has been implicated in detoxification, cell proliferation, differentiation, drug resistance, and response to oxidative stress [29, 30]. Thus, deregulation of these enzymes could result in various cancers, including esophageal squamous cell carcinoma [31] and breast cancer [32]. Giacalone et al. [33] reported that ALDH7A1, one of the ALDH superfamily members, was correlated with OS and recurrence in patients with surgically resected stage I NSCLC.

MDH1, an NAD(H)-dependent enzyme, is an important part in the malate/aspartate shuttle (MAS) [34]. This metabolic cycle contributes to maintaining intracellular NAD(H) redox homeostasis as it transfers the reducing equivalent NAD(H) across the mitochondrial membrane [34]. It has been reported that abnormal expression of MDH1 is related to tumor occurrence and progression [35]. For example, MDH1 promoted pancreatic ductal adenocarcinoma cell proliferation and metabolism through NAD production to support glycolysis [35, 36]. Zhang et al. [37] reported that MDH1 expression was elevated in NSCLC tissue compared with normal lung tissue. However, there were no combinations of these three glycolysis-related genes (HKDC1, ALDH7A1, and MDH1) to predict the prognosis of LUSC.

This study is the first to report that a glycolysis-based three-gene signature can serve as a prognostic indicator for patients with LUSC. A higher risk score indicates a worse prognosis. Of course, some study limitations remain. First, the risk score model was constructed using the TCGA database and should be verified in other cohorts in future studies. Second, studies on the three predicted genes should be performed to explore concrete mechanisms in the occurrence and development of LUSC.

## Conclusions

This study suggested that the three-gene signature associated with glycolysis might not only help to predict prognosis of LUSC patients, but also can provide additional gene targets that can be potentially used to cure LUSC patients.

## Supplementary Information

**Additional file 1: Supplementary Figure 1**. The alteration proportion for the three selected genes in patients with LUSC. Abbreviation: LUSC, lung squamous cell carcinoma.

**Additional file 2: Supplementary Table 1**. The details of clinicopathological information for 495 patients with LUSC.

## Data Availability

All TCGA related data can be obtained from the TCGA database (https://tcga-data.nci.nih.gov/).

## Abbreviations

LUSC: Lung squamous cell carcinoma
TCGA: The Cancer Genome Atlas
GSEA: Gene set enrichment analysis
HKDC1: Hexokinase domain-containing protein 1
ALDH7A1: Aldehyde dehydrogenase 7A1
MDH1: Malate dehydrogenase 1
NSCLC: Non-small cell lung cancer
SCLC: Small cell lung cancer
LUAD: Lung adenocarcinoma
OS: Overall survival
EGFR: Epidermal growth factor receptor
KNG1: Kininogen 1
TRIM59: Tripartite motif-containing protein 59
HR: Hazard ratio
ROC: Receiver operating characteristic
AUC: Area under the ROC curve
DPP9: Dipeptidyl peptidase 9
FoxQ1: Forkhead box Q1
ENO1: Enolase1
GLUT1: Glucose transporter 1
AJCC: American Joint Committee on Cancer

## References

[1] Osmani L, Askin F, Gabrielson E, Li QK (2018). Current WHO guidelines and the critical role of immunohistochemical markers in the subclassification of non-small cell lung carcinoma (NSCLC): moving from targeted therapy to immunotherapy. Semin Cancer Biol.

[2] Molinier O, Goupil F, Debieuvre D, Auliac JB, Jeandeau S, Lacroix S, Martin F, Grivaux M (2019). Five-year survival and prognostic factors according to histology in 6101 non-small-cell lung cancer patients. Respir Med Res.

[3] Pirker R (2015). What is the best strategy for targeting EGF receptors in non-small-cell lung cancer?. Future Oncol.

[4] Siegel RL, Miller KD, Jemal A (2019). Cancer statistics, 2019. CA Cancer J Clin.

[5] Hanahan D, Weinberg RA (2011). Hallmarks of cancer: the next generation. Cell..

[6] Li Z, Zhang H (2016). Reprogramming of glucose, fatty acid and amino acid metabolism for cancer progression. Cell Mol Life Sci.

[7] Lunt SY, Heiden MGV (2011). Aerobic glycolysis: meeting the metabolic requirements of cell proliferation. Annu Rev Cell Dev Biol.

[8] Wang W, Wang S, Zhang M (2020). Evaluation of kininogen 1, osteopontin and α-1-antitrypsin in plasma, bronchoalveolar lavage fluid and urine for lung squamous cell carcinoma diagnosis. Oncol Lett.

[9] Lou M, Gao Z, Zhu T, Mao X, Wang Y, Yuan K, Tong J (2020). TRIM59 as a novel molecular biomarker to predict the prognosis of patients with NSCLC. Oncol Lett.

[10] Wang X, Li G, Luo Q, Xie J, Gan C (2018). Integrated TCGA analysis implicates lncRNA CTB-193M12.5 as a prognostic factor in lung adenocarcinoma. Cancer Cell Int.

[11] Ge H, Yan Y, Wu D, Huang Y, Tian F (2018). Potential role of LINC00996 in colorectal cancer: a study based on data mining and bioinformatics. Onco Targets Ther.

[12] The Cancer Genome Atlas Research Network (2011). Integrated genomic analyses of ovarian carcinoma. Nature..

[13] The Cancer Genome Atlas Research Network (2012). Comprehensive genomic characterization of squamous cell lung cancers. Nature..

[14] Tang Z, Li J, Shen Q, Feng J, Liu H, Wang W, Xu L, Shi G, Ye X, Ge M, Zhou X, Ni S (2017). Contribution of upregulated dipeptidyl peptidase 9 (DPP9) in promoting tumoregenicity, metastasis and the prediction of poor prognosis in non-small cell lung cancer (NSCLC). Int J Cancer.

[15] Feng J, Zhang X, Zhu H, Wang X, Ni S, Huang J (2012). FoxQ1 overexpression influences poor prognosis in non-small cell lung cancer, associates with the phenomenon of EMT. PLoS One.

[16] Zhang L, Zhang Z, Yu Z (2019). Identification of a novel glycolysis-related gene signature for predicting metastasis and survival in patients with lung adenocarcinoma. J Transl Med.

[17] Liu C, Li Y, Wei M, Zhao L, Yu Y, Li G (2019). Identification of a novel glycolysis-related gene signature that can predict the survival of patients with lung adenocarcinoma. Cell Cycle.

[18] Abbaszadeh Z, Cesmeli S, Biray AC (2020). Crucial players in glycolysis: Cancer progress. Gene..

[19] Warburg O (1956). On respiratory impairment in cancer cells. Science..

[20] Koppenol WH, Bounds PL, Dang CV (2011). Otto Warburg's contributions to current concepts of cancer metabolism. Nat Rev Cancer.

[21] Fu QF, Liu Y, Fan Y, Hua SN, Qu HY, Dong SW, Li RL, Zhao MY, Zhen Y, Yu XL, Chen YY, Luo RC, Li R, Li LB, Deng XJ, Fang WY, Liu Z, Song X (2015). Alpha-enolase promotes cell glycolysis, growth, migration, and invasion in non-small cell lung cancer through FAK-mediated PI3K/AKT pathway. J Hematol Oncol.

[22] Osugi J, Yamaura T, Muto S, Okabe N, Matsumura Y, Hoshino M, Higuchi M, Suzuki H, Gotoh M (2015). Prognostic impact of the combination of glucose transporter 1 and ATP citrate lyase in node-negative patients with non-small lung cancer. Lung Cancer.

[23] Zhang B, Xie Z, Li B (2019). The clinicopathologic impacts and prognostic significance of GLUT1 expression in patients with lung cancer: a meta-analysis. Gene..

[24] Irwin DM, Tan H (2008). Molecular evolution of the vertebrate hexokinase gene family: identification of a conserved fifth vertebrate hexokinase gene. Comp Biochem Physiol Part D Genomics Proteomics.

[25] Fuhr L, El-Athman R, Scrima R, Cela O, Carbone A, Knoop H, Li Y, Hoffmann K, Laukkanen MO, Corcione F (2018). The circadian clock regulates metabolic phenotype rewiring via HKDC1 and modulates tumor progression and drug response in colorectal Cancer. EBioMedicine..

[26] Zhang Z, Huang S, Wang H, Wu J, Chen D, Peng B, Zhou Q (2016). High expression of hexokinase domain containing 1 is associated with poor prognosis and aggressive phenotype in hepatocarcinoma. Biochem Biophys Res Commun.

[27] Chen X, Lv Y, Sun Y, Zhang H, Xie W, Zhong L, Chen Q, Li M, Li L, Feng J, Yao A, Zhang Q, Huang X, Yu Z, Yao P (2019). PGC1β regulates breast tumor growth and metastasis by SREBP1-mediated HKDC1 expression. Front Oncol.

[28] Wang X, Shi B, Zhao Y, Lu Q, Fei X, Lu C, Li C, Chen H (2020). HKDC1 promotes the tumorigenesis and glycolysis in lung adenocarcinoma via regulating AMPK/mTOR signaling pathway. Cancer Cell Int.

[29] Muzio G, Maggiora M, Paiuzzi E, Oraldi M, Canuto RA (2012). Aldehyde dehydrogenases and cell proliferation. Free Radic Biol Med.

[30] Ahmed Laskar A, Younus H (2019). Aldehyde toxicity and metabolism: the role of aldehyde dehydrogenases in detoxification, drug resistance and carcinogenesis. Drug Metab Rev.

[31] Wang H, Tong L, Wei J, Pan W, Li L, Ge Y, Zhou L, Yuan Q, Zhou C, Yang M (2014). The ALDH7A1 genetic polymorphisms contribute to development of esophageal squamous cell carcinoma. Tumour Biol.

[32] Marcato P, Dean CA, Pan D, Araslanova R, Gillis M, Joshi M, Helyer L, Pan L, Leidal A, Gujar S, Giacomantonio CA, Lee PWK (2011). Aldehyde dehydrogenase activity of breast cancer stem cells is primarily due to isoform ALDH1A3 and its expression is predictive of metastasis. Stem Cells.

[33] Giacalone NJ, Den RB, Eisenberg R, Chen H, Olson SJ, Massion PP, Carbone DP, Lu B (2013). ALDH7A1 expression is associated with recurrence in patients with surgically resected non-small-cell lung carcinoma. Future Oncol.

[34] Broeks MH, Shamseldin HE, Alhashem A, Hashem M, Abdulwahab F, Alshedi T, Alobaid I, Zwartkruis F, Westland D, Fuchs S, Verhoeven-Duif NM, Jans JJM, Alkuraya FS (2019). MDH1 deficiency is a metabolic disorder of the malate-aspartate shuttle associated with early onset severe encephalopathy. Hum Genet.

[35] Hanse EA, Ruan C, Kachman M, Wang D, Lowman XH, Kelekar A (2017). Cytosolic malate dehydrogenase activity helps support glycolysis in actively proliferating cells and cancer. Oncogene..

[36] Wang YP, Zhou W, Wang J, Huang X, Zuo Y, Wang TS, Gao X, Xu YY, Zou SW, Liu Y-B, Cheng JK, Lei QY (2016). Arginine methylation of MDH1 by CARM1 inhibits glutamine metabolism and suppresses pancreatic Cancer. Mol Cell.

[37] Zhang B, Tornmalm J, Widengren J, Vakifahmetoglu-Norberg H, Norberg E (2017). Characterization of the role of the malate dehydrogenases to lung tumor cell survival. J Cancer.

