# Supplementary figures and images for: A glycolysis-based three-gene signature predicts survival in patients with lung squamous cell carcinoma
Source: BMC Cancer. 2021 May 27;21:626. doi: 10.1186/s12885-021-08360-z (PMC8161559; doi:10.1186/s12885-021-08360-z)

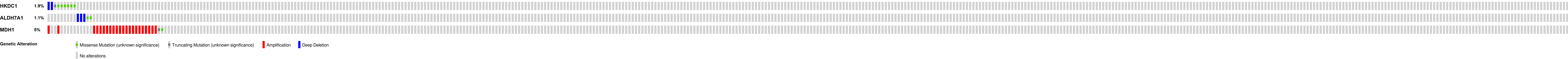

Supplement: Supplementary file 1 — Additional file 1: Supplementary Figure 1. The alteration proportion for the three selected genes in patients with LUSC. Abbreviation: LUSC, lung squamous cell carcinoma. [file 12885_2021_8360_MOESM1_ESM.tif]
