# Supplementary material for: A glycolysis-based three-gene signature predicts survival in patients with lung squamous cell carcinoma
Source: BMC Cancer. 2021 May 27;21:626. doi: 10.1186/s12885-021-08360-z (PMC8161559; doi:10.1186/s12885-021-08360-z)
Supplement: Supplementary file 2 — Additional file 2: Supplementary Table 1. The details of clinicopathological information for 495 patients with LUSC. [file 12885_2021_8360_MOESM2_ESM.docx]

**Supplementary Table 1** . The details of clinicopathological information for 495 patients with LUSC

| **Clinical characteristic** | | **N(495)** |
| --- | --- | --- |
| **Age** | ≤65 | 189 |
|  | >65 | 300 |
|  | NA | 6 |
| **Sex** | Female | 129 |
|  | Male | 366 |
|  | NA | 0 |
| **AJCC stage** | I-II | 401 |
|  | III-IV | 90 |
|  | NA | 4 |
| **T** | T1-T2 | 402 |
|  | T3-T4 | 93 |
|  | NA | 0 |
| **N** | N0 | 316 |
|  | N1–3 | 173 |
|  | NA | 6 |
| **M** | M0 | 407 |
|  | M1 | 7 |
|  | NA | 81 |
| **Survival time** | reported | 495 |
| **Survival status** | Alive | 284 |
|  | Dead | 211 |

**Abbreviations**: LUSC, lung squamous cell carcinoma; TCGA, The Cancer Genome Atlas; NA, not available; AJCC, American Joint Committee on Cancer.

**Note**: Owing to 6 of 501 patients lacked the data of survival time, a total of 495 patients were included in the survival analysis. The ID of these 6 patients was listed as follows: TCGA-63-5128, TCGA-6A-AB49, TCGA-63-5131, TCGA-56-6546, TCGA-63-A5MU, TCGA-79-5596.
